# The effects of exercise training interventions on depression in hemodialysis patients

**DOI:** 10.3389/fpsyt.2023.1321413

**Published:** 2024-01-08

**Authors:** Huihui Yu, Mei Huang, Yuxiu Tao, Shanshan Li, Jing Wang, Ping Li, Honghong Lv, Chunping Ni

**Affiliations:** ^1^School of Nursing, Air Force Medical University, Xi’an, China; ^2^The 1th Department of Gerontology, the 960th Hospital of PLA Joint Logistics Support Force, Jinan, China; ^3^School of Nursing, Shaanxi University of Chinese Medicine, Xianyang, China; ^4^Blood Purification Center, Tangdu Hospital, Air Force Medical University, Xi’an, China

**Keywords:** depression, exercise training, intradialytic exercise, hemodialysis, end-stage renal disease

## Abstract

**Purpose:**

Depression considerably influences the clinical outcomes, treatment compliance, quality of life, and mortality of hemodialysis patients. Exercise plays a beneficial role in depressive patients, but its quantitative effects remain elusive. This study aimed to summarize the effects of exercise training on depression in patients with end-stage renal disease undergoing hemodialysis.

**Methods:**

The PUBMED, EMBASE, and Cochrane Library databases were systematically searched from inception to April 2023 to identify published articles reporting the effect of exercise training on the depression level of patients with End-Stage Renal Disease undergoing hemodialysis. Data were extracted from the included studies using predefined data fields by two independent researchers. The Cochrane Handbook for Systematic Reviews of Interventions and Joanna Briggs Institute Critical Appraisal Checklist for Quasi-Experimental Studies were employed for quality evaluation.

**Results:**

A total of 22 studies enrolling 1,059 patients who participated in exercise interventions were included. Hemodialysis patients exhibited superior outcomes with intradialytic exercise (SMD = −0.80, 95% CI: −1.10 to −0.49) and lower levels of depression following aerobic exercise (SMD = −0.93, 95%CI: −1.32 to −0.55) compared to combined exercise (c − 0.85, 95% CI: −1.29 to −0.41) and resistance exercise (SMD = −0.40, 95%CI: −0.96 to 0.17). Regarding exercise duration, patients manifested lower depression levels when engaging in exercise activities for a duration exceeding 6 months (SMD = −0.92, 95% CI: −1.67 to −0.17). Concerning the duration of a single exercise session, the most significant improvement was noted when the exercise duration exceeded 60 min (SMD = −1.47, 95% CI: −1.87 to −1.06).

**Conclusion:**

Our study determined that exercise can alleviate depression symptoms in hemodialysis patients. This study established the varying impacts of different exercise parameters on the reduction of depression levels in hemodialysis patients and is anticipated to lay a theoretical reference for clinicians and nurses to devise tailored exercise strategies for interventions in patients with depression.

**Systematic review registration:**

https://www.crd.york.ac.uk/prospero/, This study was registered in the International Prospective Register of Systematic Reviews (PROSPERO) database, with registration number CRD42023434181.

## 1 Introduction

End-stage renal disease (ESRD), the fifth stage of chronic kidney disease (CKD), arises from diverse etiologies and is characterized by low quality of life and high mortality rates. As is well documented, the most effective treatment modality for ESRD remains hemodialysis (HD) (1). Recent clinical research estimated that nearly 5 million ESRD patients will necessitate HD as a kidney replacement therapy worldwide by 2030 (2). While considerable advancements have been made in the treatment and care of hemodialysis patients, the long-term prognosis of these patients remains dismal (3).

The prevalence of depression in patients with HD is a particular concern. Indeed, depressive symptoms afflict approximately 74.58% of patients undergoing HD and are associated with suicidal tendencies, lower quality of life, reduced treatment compliance, medical comorbidities, and elevated mortality rates (4–8). Therefore, effective nursing interventions are urgently warranted to aid patients in coping with depression. At present, the primary approaches for the treatment of depression involve a combination of pharmacological and non-pharmacological interventions. Nonetheless, the majority of antidepressants elicit adverse events, such as drug dependence, addiction, and poor tolerance (9). A previous study demonstrated promising outcomes for non-pharmacological interventions, specifically the incorporation of exercise training as a supplement to pharmacotherapy (10).

The National Kidney Function Association recommends Exercise training as a cornerstone for HD patients to effectively manage complications (11). A recent study documented that exercise training could assist HD patients in achieving a better quality of life and could significantly alleviate depressive symptoms (12). However, given that the intervention is affected by specific training parameters, including the type of exercise, the location of exercise, the duration and the frequency of exercise, and so on, a comprehensive standard of exercise has not been established so far. While earlier studies focused on the effects of exercise on physical parameters such as blood pressure, motor function, and hemoglobin levels in patients with ESRD (12, 13), those targeting mental parameters are scarce, and the findings are often contradictory. Therefore, the purpose of this systematic review and meta-analysis was to aggregate available evidence and systematically analyze the effects of exercise on depression in HD patients to assess the effectiveness of different exercise training parameters.

## 2 Methods

This study was performed in accordance with the PRISMA guidelines for systematic reviews and meta-analyses. This systematic review was conducted as a quantitative systematic review and meta-analysis utilizing the fixed-effects model (14). This study was registered in the International Prospective Register of Systematic Reviews (PROSPERO) database, with registration number CRD42023434181.

### 2.1 Data sources and searches

A systematic search was performed in the PUBMED, EMBASE, and Cochrane Library databases to screen for published articles from database inception to April 2023 with a combination of medical subject headings (MeSH) terms and text keywords. Key words included in the search were hemodialysis, mental disorder, depressive disorder, stress disorders, and exercise (see details in Supplementary Methods 1). In addition, the scope of our search was expanded to manually search the reference list of original studies, as well as grey literature and records.

### 2.2 Inclusion and exclusion criteria

Inclusion criteria for this study were as follows: (1) population: adult ≥18 years of age with a diagnosis of ESRD requiring HD; (2) study design: both quasi-randomized controlled trials and clinical randomized controlled trials; (3) intervention and comparison: exercise training of intervention group comprised aerobic training, resistance training, and combined training, while the control group received usual care, sham exercise, and no exercise. (4) Outcomes: the primary end-point was depression. Assessment tools for depression included the Beck Depression Inventory, the self-rating depression scale developed by Zung, the Hospital Anxiety and Depressive Depression Scale, etc. Exclusion criteria were as follows: (1) Inappropriate study types, such as reviews, editorials, and case reports; (2) patients undergoing alternative renal replacement therapies or those suffering from acute kidney failure. (3) Studies with incomplete data.

### 2.3 Quality assessment

The quality of all the included randomized controlled trial studies was assessed following the guidelines outlined in the Cochrane Handbook for Systematic Reviews of Interventions. Seven aspects were considered, namely random generation, allocation concealment, blinding of participants and outcome assessment, incomplete outcome data, selective reporting, and other biases. All included trials were independently assessed, and the risk of bias was classified as “low risk,” “unclear,” or “high risk” (15). The Joanna Briggs Institute (JBI) Critical Appraisal Checklist for Quasi-Experimental Studies was used for quality evaluation of non-randomized experimental studies, which involved the consideration of 9 aspects, with each item categorized as “Yes,” “No,” “Unclear,” or “Not Applicable” (16). Disagreements between the 2 reviewers (HY and MH) were resolved by a third reviewer (YT).

### 2.4 Data extraction

The literature search, study selection, study appraisal, and data extraction were pre-defined in the protocol and independently conducted by two investigators (HY and MH). Data extracted for this study included the following: first author, publication year, country, study design, sample size, male-to-female ratio, mean age, intervention (exercise type, exercise location, total exercise duration, single exercise duration, and outcome measurement tools). Discrepancies between the two reviewers were resolved by discussion until reaching a consensus.

### 2.5 Data synthesis and statistical analysis

Statistical analyses were performed using Review Manager 5.3 (version 5.3.5, Copenhagen: The Nordic Cochrane Center, The Cochrane Collaboration) and Comprehensive Meta-Analysis Version 3.0 software (Biostat, Englewood, NJ), and *p* < 0.05 was considered statistically significant. Considering that the studies used different measurement scales to measure depression levels, the standardized differences in mean (SMD), along with their 95% confidence intervals (CI), were used to estimate the intervention effect size. The Tau^2^ and *I*^2^ statistics were used to assess heterogeneity among studies. *I*^2^ values below 25% indicated low heterogeneity, values between 25 and 50% suggested moderate heterogeneity and values exceeding 50% indicated high heterogeneity. If the heterogeneity was high, subgroup analysis was performed, and the random effects model was used. To assess the potential presence of publication bias, the symmetry of the funnel chart was examined. Sensitivity analyses were performed by excluding each study one at a time and comparing the results with the original findings.

## 3 Results

### 3.1 Search outcome

**Figure 1:**
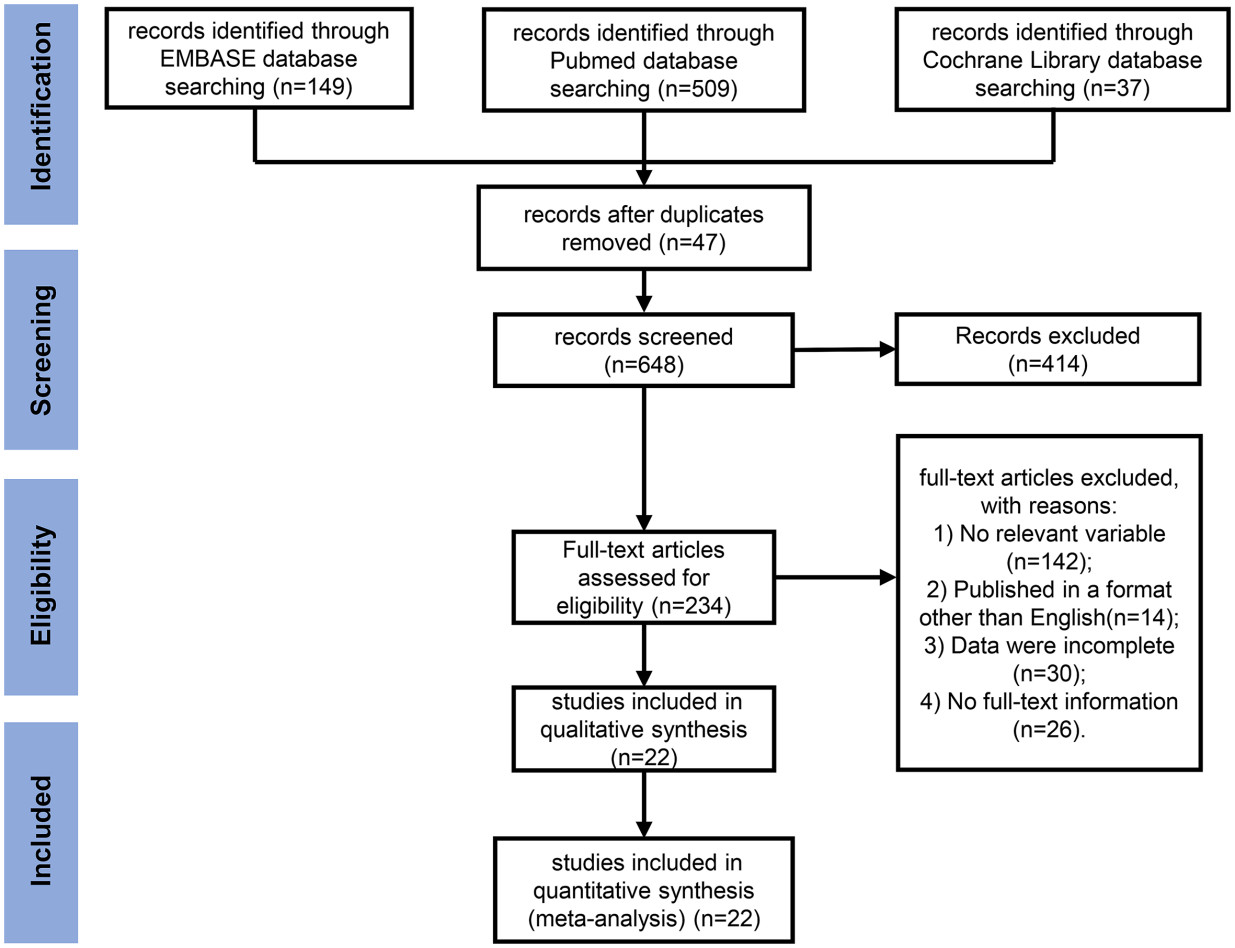
PRISMA flow diagram for the meta-analysis. A flow diagram depicting the studies screened, assessed for eligibility, and included in the review, along with reasons for exclusions.

Two reviewers (H.Y. and M.H.) independently searched the titles and abstracts of the aforementioned databases using a standardized form. Discrepancies between the two reviewers were resolved by discussion until a consensus was attained. The study selection process is illustrated in the Preferred Reporting Items for Systematic Reviews and Meta-Analyses flow diagram (shown in Figure. 1). The database search yielded a total of 695 original studies. Following the removal of duplicates, 648 studies remained. These articles were screened by reviewers, which led to the exclusion of studies with no relevant variables (*n* = 142), publications in non-English languages (*n* = 14), incomplete data (*n* = 30), and those lacking full-text information (*n* = 26). A total of 22 articles were retained after the full-text screening process and manual search of the reference list of the full-text articles. A systematic review was performed, adhering to Preferred Reporting Items for Systematic Reviews and Meta-Analyses (PRISMA) guidelines (Supplementary Table 1).

### 3.2 Study selection and baseline characteristics

According to the inclusion and exclusion criteria, a total of 22 studies were included for analysis. The detailed characteristics of these studies are listed in Table 1. A total of 1,059 patients were included in this study. Furthermore, all the studies were published in the English language from 1983 to 2023, and were conducted in China (*n* = 4) (17–20), United States (*n* = 3) (21–23), Greece (*n* = 5) (24–28), Japan (*n* = 1) (29), Brazil (*n* = 1) (30), Spain (*n* = 1) (31), Poland (*n* = 1) (32), Iran (*n* = 2) (33, 34), Korea (*n* = 1) (35), Netherlands (*n* = 2) (36), New Zealand (*n* = 1) (37) and Turkey (*n* = 1) (38). Sixteen studies were randomized controlled trials, and 6 studies were quasi-experimental trials.

**Table 1:** Characteristics of studies and patients included in the meta-analysis.

Author, year	Country	study type	Sample size, n(male/total)		Age, years, mean ± SD		type	duration	Single duration	timing	Measuring tools
			intervention	control	intervention	control					
Liu et al. (17)	China	RCT	6/10	5/10	44.3 ± 6.7	33.2 ± 7.0	AE	12 weeks	30 min	Intradialytic	BDI-II
Tang et al. (18)	China	RCT	28/42	23/42	46.26 ± 15.61	43.90 ± 12.44	AE	12 weeks	20-30MIN	non-intradialytic	HAD-D
Lin et al. (19)	China	RCT	22/32	19/32	62.0 ± 9.5	62.1 ± 12.3	AE	12 weeks	30MIN	Intradialytic	BDI
Zhou et al. (20)	China	nRCT	35/79	/	55.03 ± 10.69	/	CE	12 weeks	30MIN	Intradialytic	HADS
Carney et al. (21)	USA	nRCT	4	4	38.75 ± 8.02	43.25 ± 7.14	AE	6 months	/	non-intradialytic	MAACL
Carney et al. (22)	USA	RCT	5/10	3/7	36.1 ± 3.2	40.7 ± 5.3	AE	6 months	45-60MIN	Intradialytic	BDI
Zhou et al. (23)	USA	nRCT	18/37	22/36	62.7 ± 6.8	66.5 ± 10.0	CE	4 weeks	30MIN	Intradialytic	CES-D
Kouidi et al. (24)	Greece	RCT	11/20	4/11	49.6 ± 12.1	52.8 ± 10.2	CE	6 months	90MIN	non-intradialytic	BDI
Sakkas et al. (25)	Greece	RCT	5/7	5/7	48 ± 14	70 ± 11	AE	16 weeks	45MIN	intradialytic	Zung
Ouzouni et al. (26)	Greece	RCT	14/19	13/14	47.4 ± 15.7	50.5 ± 11.7	CE	10 months	60-90 min	Intradialytic	BDI
Kouidi et al. (27)	Greece	RCT	14/24	12/20	46.3 ± 11.2	45.8 ± 10.9	CE	12 months	60-90MIN	Intradialytic	BDI; HADS
Giannaki et al. (28)	Greece	RCT	11/15	5/7	56.4 ± 12.5	56.8 ± 16.5	AE	6 months	45MIN	non-intradialytic	Zung
Yabe et al. (29)	Japan	RCT	14/27	15/19	75.3 ± 4.4	74.3 ± 5.8	CE	12 months	30MIN	Intradialytic	GDS
Deus et al. (30)	Brazilian	RCT	46/81	40/76	67.27 ± 3.24	66.33 ± 3.88	RT	6 months	60MIN	Predialysis	BDI
Ortega-Pérez de Villar et al. (31)	Spain	RCT	15/24	14/22	62.2 ± 15.0	59.3 ± 16.1	AE	16 weeks	20-40MIN	Predialysis	CES-D
Dziubek et al. (32)	Poland	RCT	9/20	5/8	66.3 ± 13.1	56.4 ± 13.6	CE	6 months	50 min	Predialysis	BDI
Rezaei et al. (33)	Iran	nRCT	21/25	14/26	43.96 ± 7.86	42.61 ± 12.67	CE	10 weeks	35MIN	non-intradialytic	BDI
Rahimimoghadam et al. (34)	Iran	RCT	21/25	20/25	39.1 ± 2.2	38.4 ± 1.8	AE	8 weeks	45MIN	non-intradialytic	GHQ-28
Rhee et al. (35)	Korea	nRCT	9/22	/	57.0 ± 12.4	/	CE	6 months	40-50 min	Intradialytic	BDI
van Vilsteren et al. (36)	The Netherlands	RCT	33/53	30/43	52 ± 15	58 ± 16	CE	12 weeks	40MIN	Predialysis	SDS
Cheema et al. (37)	New Zealand	RCT	17/24	17/25	60.0 ± 15.3	65.0 ± 12.9	RT	12 weeks	/	Intradialytic	GDS
Levendoğlu et al. (38)	Turkey	nRCT	8/14	/	33.1 ± 13.1	/	AE	12 weeks	90MIN	non-intradialytic	BDI

AE, aerobic exercise; CE, combined exercise; RT, resistance training; BDI, Beck Depression Inventory; Zung, the self-rating depression scale developed by Zung; HADS, Hospital Anxiety and Depressive Depression Scale; CES-D, Center for Epidemiological Studies Depression; GDS, Geriatric Depression Scale; SDS, The 20-item Self-rating Depression Scale; GHQ-28, general health questionnaire-28; MAACL, Multiple Affect Adjective Checklist; /, unclear information.

Of the 22 studies, 11 involved intradialytic exercises, 11 examined non-intradialytic exercises, and four explored pre-dialytic exercises. All interventions were delivered on a regular basis, with the intervention duration ranging from 1 month to 12 months. The majority of exercise interventions consisted of combining 2 or 3 types of training (*n* = 6); the remaining studies primarily focused on aerobic exercise (*n* = 9) and resistance exercise (*n* = 1). In addition, exercise frequency was largely 3 times a week (*n* = 14), with two studies employing a twice-weekly regimen. Single exercise duration ranged from 20 min to 90 min. The intensity of exercise was typically moderate, with parameters such as 55 ~ 70% of the peak power or a rating of perceived exertion on the Borg scale ranging between 11 ~ 15.

### 3.3 Assessment of risks of bias

The risk of bias was principally high or uncertain due to incomplete descriptions of the methodology.

**Figure 2:**
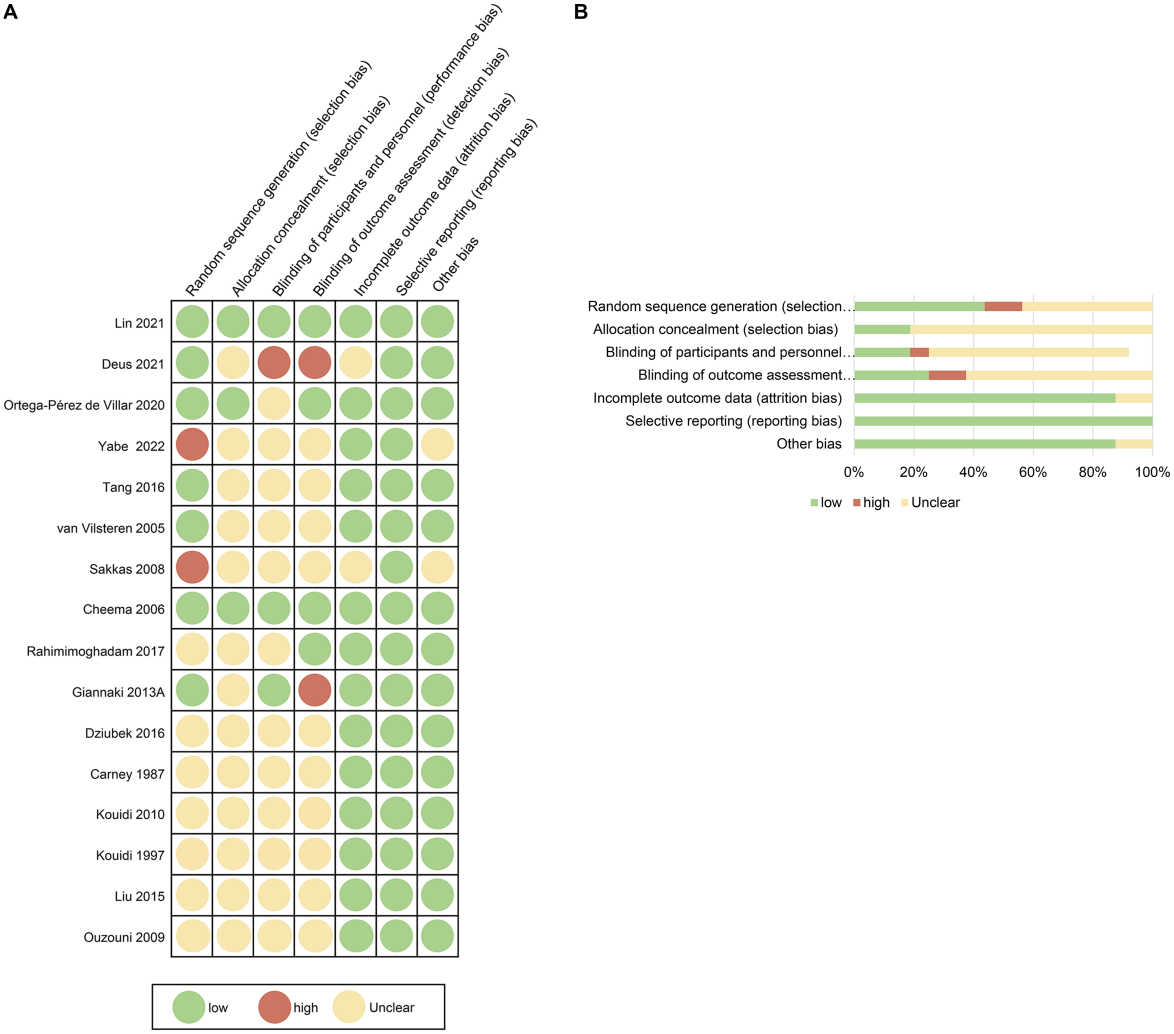
Risk of bias summary for the included studies. The methodological quality of included studies was assessed using the Cochrane Handbook for Systematic Reviews of Interventions, considering 7 aspects, namely random generation, allocation concealment, blinding of participants and outcome assessment, incomplete outcome data, selective reporting, and other biases.

Out of 7 RCT checklist items, every study reported the use of randomization; notwithstanding, merely 7 (43.75%) studies provided details on the generation of random sequencing, and 2 studies demonstrated proper allocation concealment. Considering that blinding both participants and investigators in an exercise intervention is challenging, only 3 studies blinded participants, and 2 studies employed blinding for outcome assessment. Most trials (87.5%) reported attrition. All 16 studies had complete data (Figure 2; Supplementary Table 2).

Out of 9 checklist items for the 6 quasi-experimental studies, a control group was established in 3 of the 6 studies. For the remaining 8 checklist items, the answers were all “yes” (Table 2).

**Table 2:** Quality assessment of quasi-experimental studies using the Joanna Briggs Institute (JBI) Appraisal Checklist.

Study	1. Is it clear in the study what is the ‘cause’ and what is the ‘effect’	2. Were the participants included in any comparisons similar?	3. Were the participants included in any comparisons receiving similar treatment/care, other than the exposure or intervention of interest?	4. Was there a control group?	5. Were there multiple measurements of the outcome both pre and post the intervention/exposure?	6. Was follow up complete and if not, were differences between groups in terms of their follow up adequately described and analyzed?	7. Were the outcomes of participants included in any comparisons measured in the same way?	8. Were outcomes measured in a reliable way?	9. Was appropriate statistical analysis used?
Levendoğlu 2004	yes	yes	yes	no	yes	yes	yes	yes	yes
Rhee 2019	yes	yes	yes	no	yes	yes	yes	yes	yes
Zhou 2023	yes	yes	yes	no	yes	yes	yes	yes	yes
Zhou 2020	yes	yes	yes	yes	yes	yes	yes	yes	yes
Carney 1987	yes	yes	yes	yes	yes	yes	yes	yes	yes
Rezaei 2015	yes	yes	yes	yes	yes	yes	yes	yes	yes

### 3.4 Effect of exercise on depression in HD patients

**Figure 3:**
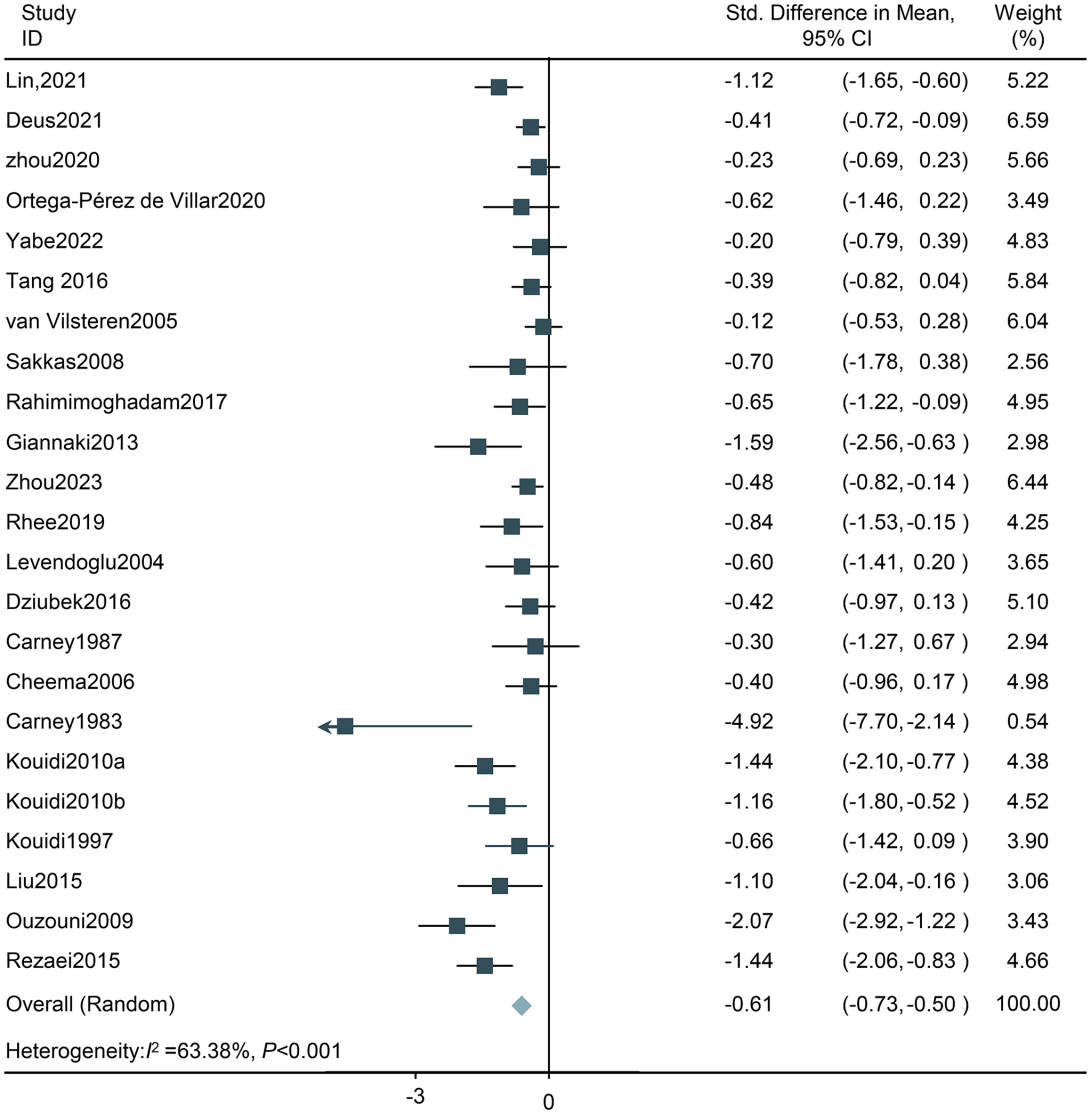
Forest plot of the effect of exercise on depression in HD patients. CI, confidence intervals.

**Total effect on depression:** Among the 22 studies, the results indicated that the intervention group had a 0.61 lower risk of experiencing depression compared with the comparison group (SMD, −0.61; 95% CI, −0.73 to −0.50; Figure 3) The analysis revealed high heterogeneity among the 22 studies (Tau^2^ = 0.15, *df* = 22; *I^2^* = 63.38%; *Z* = 10.13; *p* < 0.001). Overall, interventions in HD patients significantly decreased depression levels. Of note, sensitivity analysis yielded similar results even when each study was removed one by one. (Supplementary Figure 1).

**Figure 4:**
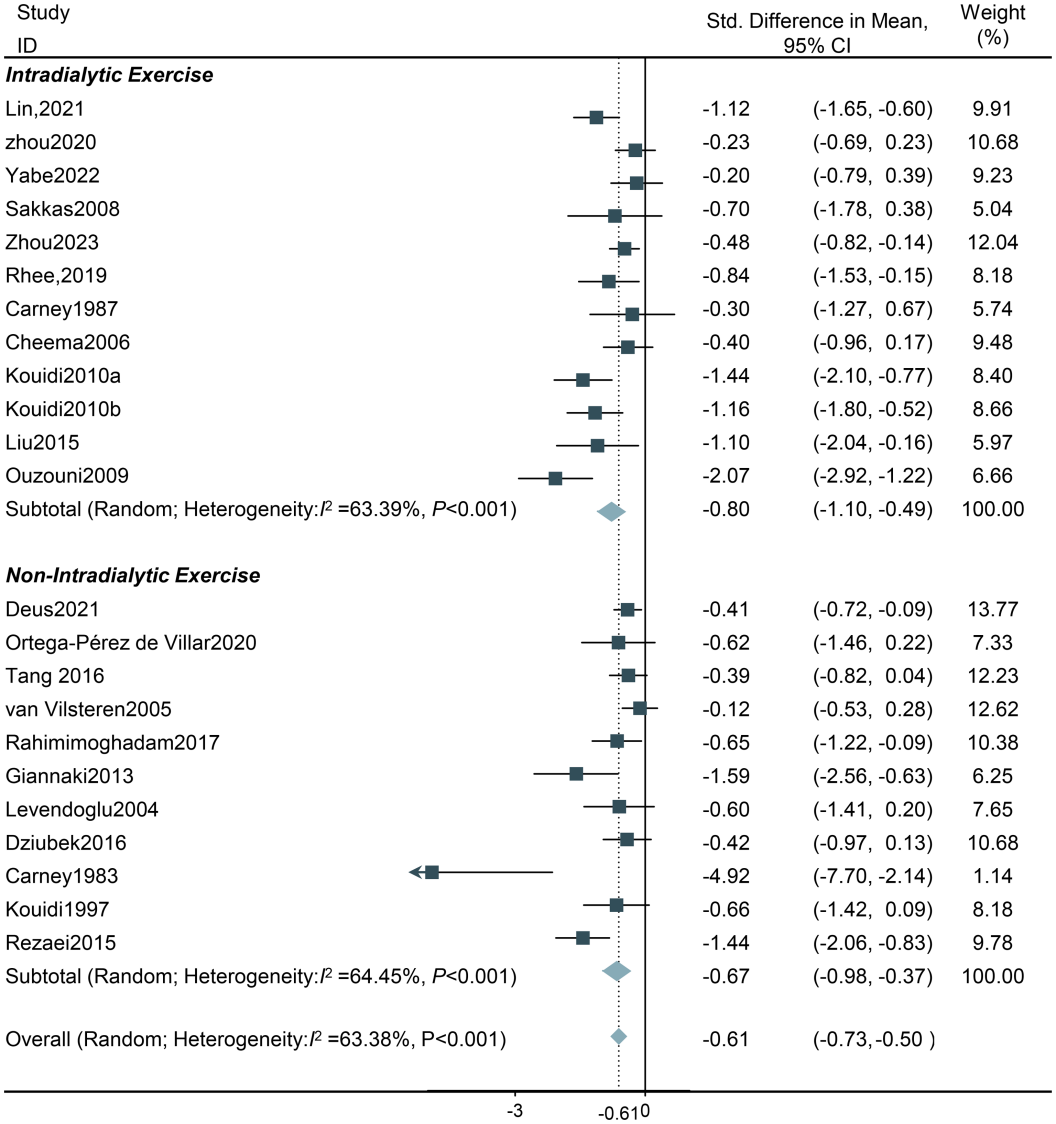
Forest plot of the effect of different exercise types on depression in HD patients. CI, confidence intervals.

**Exercise training location:** A total of 11 studies used intradialytic exercise interventions, and 11 studies used non-intradialytic exercise interventions. Both intradialytic exercise and non-intradialytic exercise intervention decreased the level of depression (SMD −0.80, 95%CI −1.10 to −0.49; SMD −0.67, 95%CI −0.98 to −0.37; respectively); nevertheless, heterogeneity was identified to be high (Tau^2^ = 0.17, *df* = 11, *I^2^* = 63.40%, *Z* = −5.17, *p* < 0.001; Tau^2^ = 0.15, *df* = 10, *I*^2^ = 64.45%, *Z* = −4.31, *p* < 0.001; respectively). Thus, the random effects model was adopted (Figure 4).

**Figure 5:**
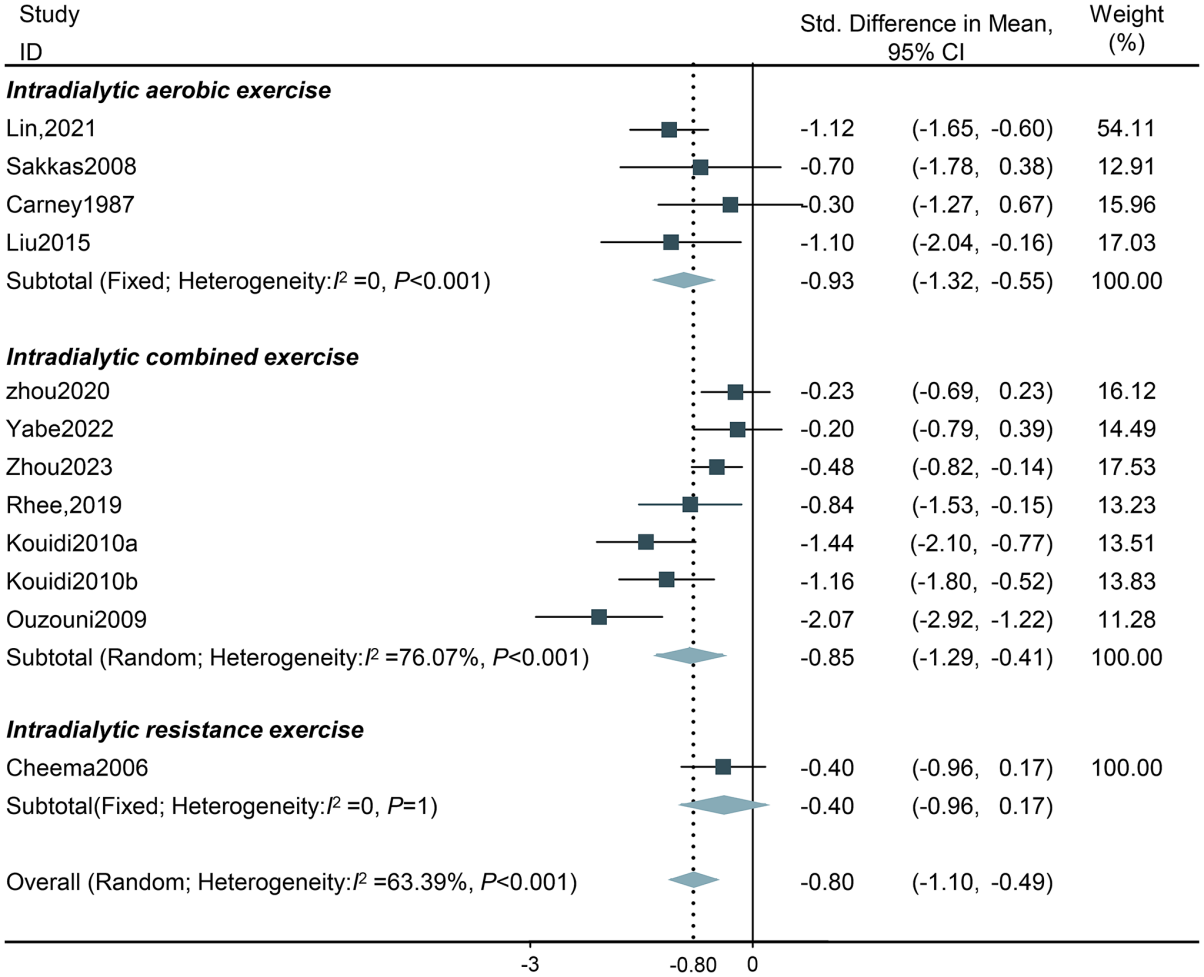
Forest plot of the effect of different exercise locations on depression in HD patients. CI, confidence intervals.

**Exercise training types:** The data revealed that exercise interventions yielded a decrease in the depression level of all four subgroups, with aerobic exercise displaying superior outcomes (SMD −0.93, 95%CI −1.32 to −0.55). Nevertheless, heterogeneity was high in the combined exercise group (Tau^2^ = 0.25, *df* = 6; *I*^2^ = 76.07%, Z = −3.79, *p* < 0.001), but all 7 studies had a total SMD < 0, and the 95% CI for SMD did not cross the equivalence line, showing a significant and positive effect. The SMD of a study administering resistance exercise intervention was −0.40, but the 95% CI crossed the equivalence line; hence, the result was not statistically significant (Figure 5).

**Figure 6:**
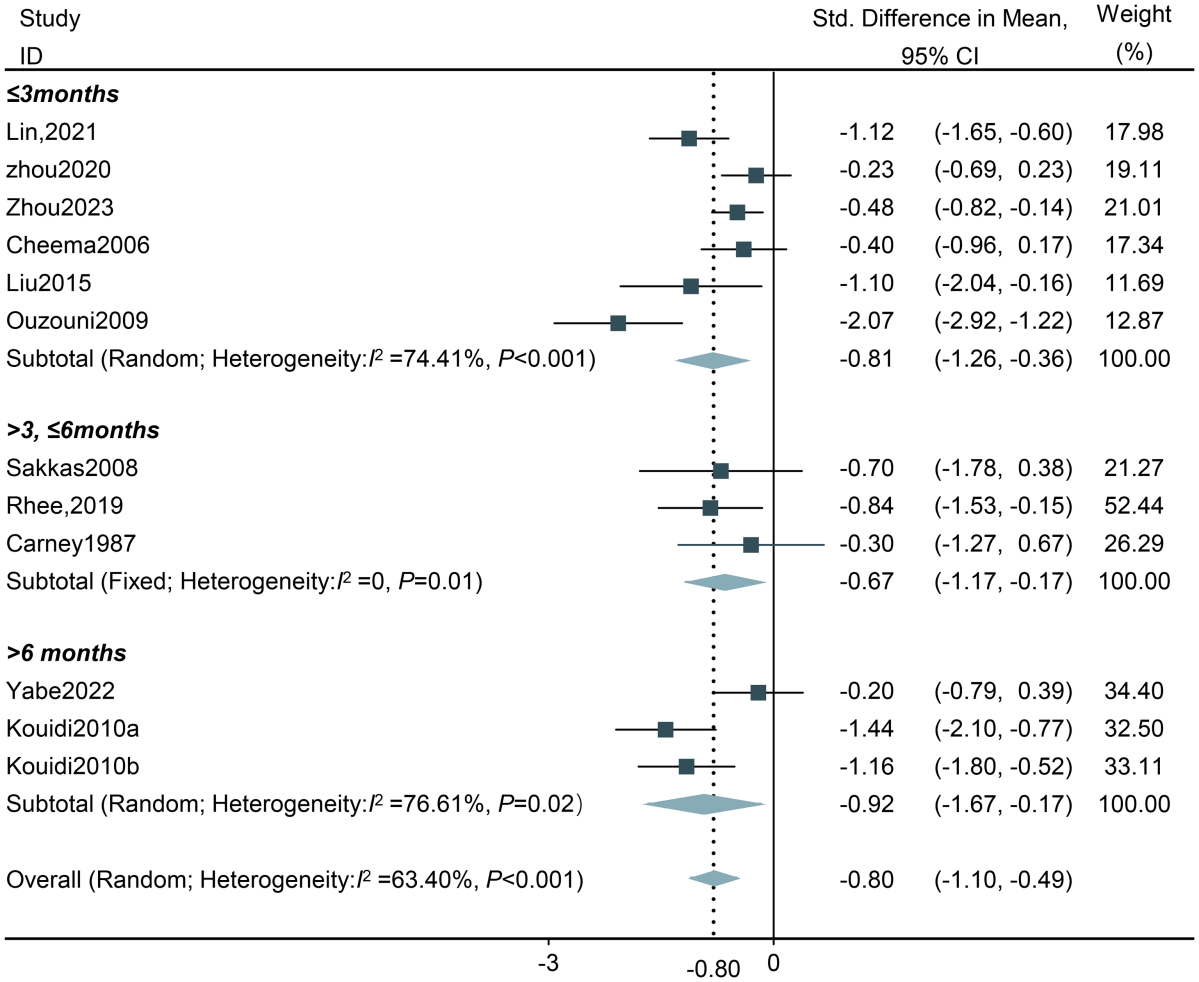
Forest plot of the effect of different total exercise duration on depression in HD patients. CI, confidence intervals.

**Exercise training duration:** The effect of intradialytic exercise on depressive symptoms exhibited a U-shape pattern, improving with prolonged follow-up, with the optimal effect observed after more than 6 months (SMD −0.81, 95%CI −1.26 to −0.36; SMD −0.67, 95%CI −1.17 to −0.17; SMD −0.92, 95%CI −1.67 to −0.17; respectively). Given that subgroup analysis comparing durations <3 months and > 6 months revealing high heterogeneity (Tau^2^ = 0.22, *df* = 5, *I^2^* = 74.41%, *Z* = −3.53, *p* < 0.001; Tau^2^ = 0.34, *df* = 2, *I*^2^ = 76.64%, *Z* = −2.39, *p* < 0.001; respectively), the random effects model was applied (Figure 6).

**Figure 7:**
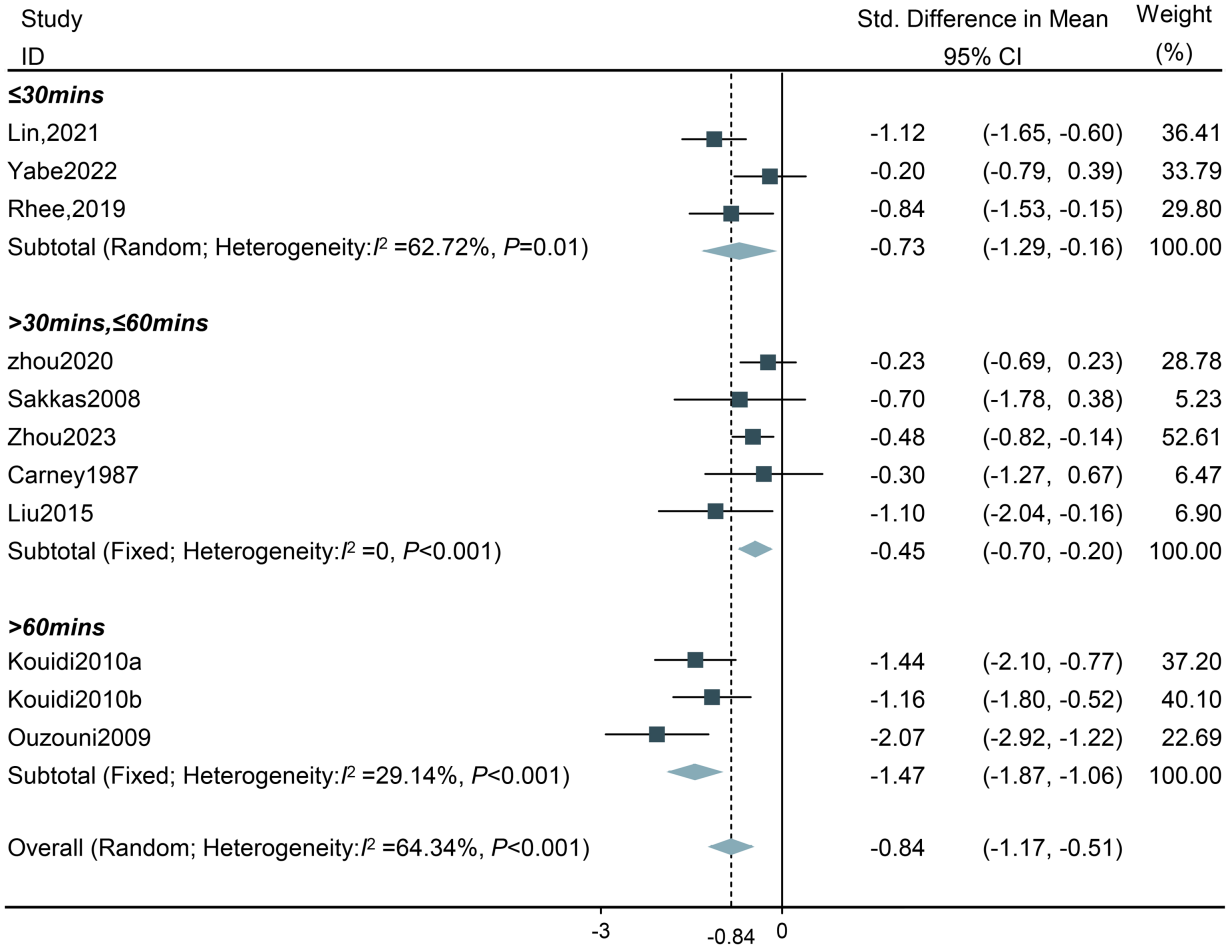
Forest plot of the effect of different single exercise durations on depression in HD patients. CI, confidence intervals.

**Single exercise training duration:** The results of the subgroup analysis demonstrated that all five subgroups with varying single exercise training durations resulted in a decrease in the depression levels of HD patients. Among them, the optimal single duration of intradialytic exercise was more than 60 min (SMD −1.47, 95%CI −1.87 to −1.06). There was a high heterogeneity in the group of less than 30 min (Tau^2^ = 0.16, *df* = 2, *I*^2^ = 62.72%, *Z* = −2.52, *p* = 0.01), and thus the random effect model was adopted (Figure 7).

### 3.5 Publication bias

**Figure 8:**
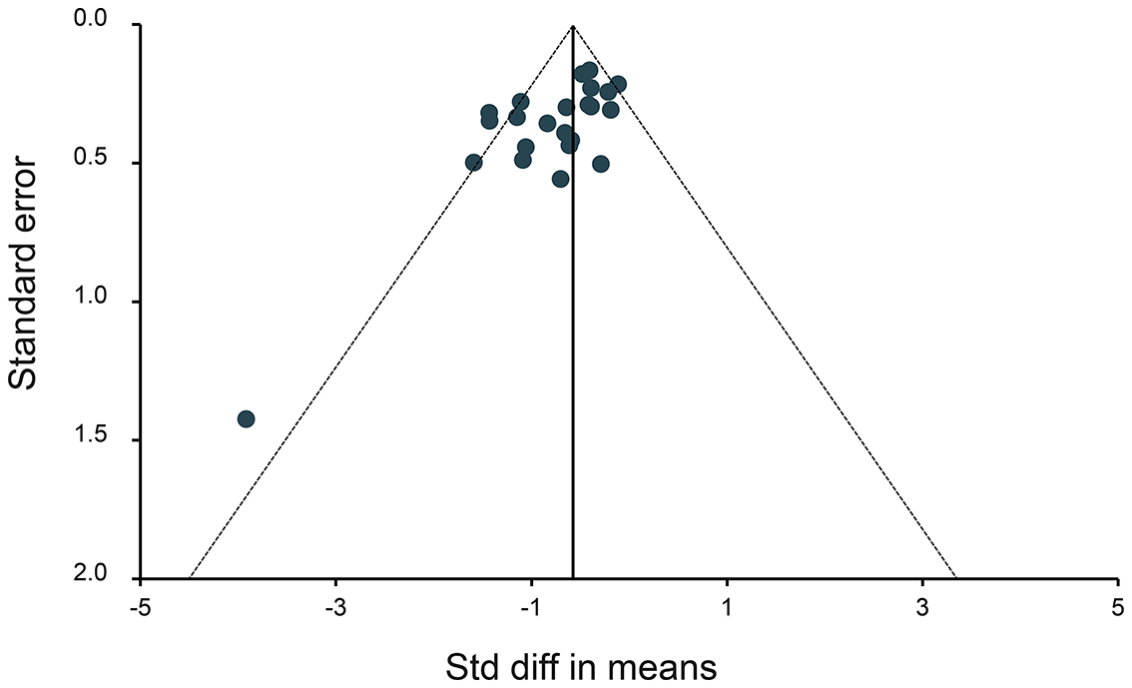
Funnel plot assessing publication bias. Data points represent individual studies. The y-axis represents the measurement of study precision (plotted as the standard error of effect size), while the x-axis represents the Std difference in means for each study. Dashed triangular lines represent the region in which 95% of studies are expected to fall in the absence of bias and heterogeneity.

The funnel plots for all studies are depicted in Figure 8. No evidence of publication bias was identified.

## 4 Discussion

This review and meta-analysis incorporated data from 1,059 HD patients and validated the benefits of intradialytic exercise to reduce the depression level of patients. This is consistent with the findings of previous studies that evinced that exercise training exerts a positive effect on depressive symptoms in HD patients (39). In this large-scale meta-analysis, in addition to calculating the total effect size of exercise training on depression levels of HD patients, the impact of different exercise parameters, including exercise location, exercise type, total exercise duration, and single exercise duration, was also assessed.

The location of exercise in HD treatment is a key factor affecting the improvement of depression in HD patients. Our meta-analysis showed that intradialytic exercise was better than non-intradialytic exercise in reducing depression levels in patients. Indeed, intradialytic exercise has been shown to improve safety and peak oxygen consumption, hemoglobin levels, and the physical component of the quality of life (40). It is typically supervised and guided by medical staff to enhance patient compliance and clinical outcomes (41). Moreover, intradialytic exercise is an effective method for utilizing the available time during hemodialysis in various countries. However, intradialytic exercise requires additional investments in equipment (such as bicycles) and increased attention from medical staff. Identifying the optimal balance between cost and cost-effectiveness warrants further exploration in future multi-center, large-scale clinical trials comparing intradialytic exercise with non-intradialytic exercise. Our study comprehensively included studies from various countries, including five from developed countries (United States, Greece, Japan, and South Korea) and two from developing countries (China). This inclusiveness partially compensates for the lack of standards and geographical differences that may result in deviations.

Regarding exercise type, comparisons between intradialytic aerobic exercise, intradialytic resistance exercise, and intradialytic combined exercise uncovered that aerobic exercise was superior in reducing depression levels compared to combined exercise and resistance exercise. A recent review pointed out that aerobic exercise could effectively enhance the cardiopulmonary function of patients with chronic kidney disease (42). The positive emotions and the well-being of patients with depression could be reinforced by good physical function (43), which is in agreement with our research results. This finding also signals that promoting intradialytic aerobic exercise could bring additional benefits to hemodialysis patients. In addition, studies have shown that simple resistance exercise is also effective in reducing the level of depression in patients. However, only one study in the subgroup of resistance exercise analysis met the inclusion criteria for our meta-analysis, and thus, the conclusions should be interpreted with caution.

The results of our analysis showed that the effectiveness of intradialytic exercise in reducing depression levels decreased between 3 and 6 months but subsequently became most significant after more than 6 months. This is inconsistent with the conclusions of previous studies that found that exercise interventions reduced depression in patients, but the effectiveness of exercise decreased over time (44). This discrepancy may be ascribed to the following reasons: patient compliance may fluctuate during the interventions. A prior investigation described that as the intervention duration increases, the exercise compliance of patients decreases (45). Notwithstanding, the compliance rate was consistently high during HD, owing to the supervision of the nursing staff. Secondly, given that participants were not blinded to exercise training and participants expected improvement in symptoms, the expected effect may wane over longer periods of time after experiencing short-term improvements. Although the effect of exercise on reducing depression levels varies, it remains effective overall. Thus, long-term exercise is recommended.

Furthermore, the duration of a single exercise session is also a crucial indicator correlated with the effectiveness of exercise (46). The duration of exercise ranged from 20 to 90 min in the included studies. Our subgroup analysis exposed that the duration of exercise in all subgroups significantly lowered the depression level in hemodialysis patients, with intradialytic exercise durations exceeding 60 min being the most effective. Paul T. Williams (47) conducted a study on the dose–response relationship between physical activity and mortality and highlighted the significant health benefits associated with exceeding the current exercise recommendations for optimal health (≥750 MET minutes per week or ≥ 1.8 MET-hours/d) as opposed to meeting them (450 to 750 MET minutes per week). Therefore, we advocate for a duration of exercise exceeding 60 min.

## 5 Study limitations

The current analysis has some limitations that merit acknowledgment. To begin, the number of studies included in each category of the exercise training group was limited. Nevertheless, publication bias assessment and sensitivity analysis showed that our results are credible. Secondly, the majority of trials on exercise interventions in patients with ESRD undergoing HD were excluded from this study during the screening phase on account that they did not fulfill the predefined inclusion criteria. This underscores the need for future RCTs to enable high-quality comparisons in order to establish the effects of exercise interventions in this patient population. Lastly, there is a paucity of eligible studies with long-term intervention durations, especially those exceeding 6 months. Therefore, the effects of long-term exercise interventions necessitate further investigation.

## 6 Conclusion

This systematic review determined that exercise training can reduce depression levels in HD patients. Clinical staff can administer extended intradialytic aerobic exercise training to ESRD patients undergoing HD and control the duration of individual exercise interventions to facilitate the recovery of patients from depression. Briefly, we recommend that HD patients with depression perform intradialytic aerobic exercise for at least 60 min in a single session and continue at least 6 months. Additionally, the desired exercise intervention team consists of multidisciplinary staff members, including clinicians, nurses, physiotherapists and occupational therapists. Nurses play a crucial role in the field of exercise prescription by assessing and monitoring patients’ physical and mental health status, collaborating with physiotherapists and occupational therapists, ensuring patient safety during exercise sessions, coordinating various aspects of patient care, providing education to patients on the importance of exercise and encouraging patients to participate in exercise actively. Cooperation among nurses, physiotherapists and occupational therapists helps to ensure that patients receive the best possible treatment and speed up the recovery process. The favorable outcomes resulting from exercise intervention on depression, physical ability, and quality of life in dialysis patients may play a positive role in elevating survival rates and concurrently decreasing hospitalization rates (48). This hypothesis necessitates further validation in future clinical trials. Furthermore, for the establishment of a patient exercise system, future studies should prioritize high-quality evidence and diversify available strategies to tailor to the specific needs of individual patients.

## Data availability statement

The raw data supporting the conclusions of this article will be made available by the authors, without undue reservation.

## Author contributions

HY: Writing – original draft, Data curation, Methodology, Conceptualization. MH: Writing – review & editing, Formal analysis, Software. YT: Data curation, Writing – review & editing. SL: Data curation, Writing – review & editing. JW: Writing – review & editing, Data curation. PL: Formal analysis, Writing – review & editing. HL: Resources, Supervision, Writing – review & editing. CN: Conceptualization, Funding acquisition, Resources, Supervision, Writing – review & editing.
